# Rapid Large-Scale Deployment of Tuberculosis Testing in a High School — Riverside County, California, 2013–2014

**Published:** 2015-06-05

**Authors:** Cameron Kaiser, Barbara Cole, Kimberly Saruwatari, Ramon Leon

**Affiliations:** 1 County of Riverside Department of Public Health, Riverside, California

*Mycobacterium tuberculosis*, the causative agent of tuberculosis (TB), can spread from person to person through the air, which can make contact investigations particularly complex in heavily populated settings such as schools. In November 2013, a student (the index patient) at a southern California high school with approximately 2,000 students and staff members was diagnosed with active pulmonary TB. Because of an unexpectedly high number of positive tuberculin skin test results in the initial contact investigation, testing was extended to the entire school population, which had to be completed before the end of the school term. A total of 1,806 persons were tested in 24 hours. The rapid testing of the entire population of a high school is unusual and led to widespread media attention and community concern, requiring close coordination among branches of the County of Riverside Department of Public Health, local governments, and the school district. The testing resulted in identification of two additional cases of TB; in addition, 72 persons underwent treatment for latent TB infection (LTBI). This incident demonstrates the importance of a coordinated emergency response in a large-scale deployment of rapid testing, including efficiently focused resources, organized testing operations, and effective media relations.

In November 2013, a student aged 14 years in Riverside County was hospitalized with active TB after a multiple-week history of cough, fever, night sweats, and 6-pound (2.7 kg) weight loss. The patient had no notable medical history, was born in the United States, and had a previously nonreactive tuberculin skin test. Upon admission, a radiograph demonstrated cavitary lung disease, and sputum was smear-positive for acid-fast bacilli. The standard four-drug TB treatment with isoniazid, rifampin, ethambutol, and pyrazinamide was promptly initiated and respiratory isolation was maintained from December 18, 2013, to January 8, 2014, when the patient was smear-negative and had received sufficient doses of antimicrobials. *M. tuberculosis* was subsequently identified by culture and was sensitive to all four first-line TB medications; genotyping was not performed. The case was reported to the county public health department, which initiated a contact investigation. The patient was subsequently discharged to continue treatment with the health department’s TB clinic. Further investigation determined that a previously known TB patient had been the index patient’s babysitter, but that potential exposure had not been reported to the health department.

## Initial Contact Investigation

In addition to contact investigation within the index patient’s family, investigation of potential contacts at the school required substantial effort because of the index patient’s multiple classes. After comparison of class records, the health department’s disease control branch identified and performed tuberculin skin testing on 198 persons at an on-campus clinic on December 16, 2013. Of this initial cohort, 59 (29.8%) were positive with an induration >5mm, and all were referred for a chest radiograph. Several preliminary results became available that evening, some with granulomatous disease and one with a large, potentially cavitary mass.

The findings were reviewed the same day by the county public health officer, who determined that sustained transmission could not be ruled out. This necessitated rapid decision-making because the school’s winter break was imminent. Only 2 days remained until the end of the school term, and there was concern that students and staff would not be accessible for testing.

## Incident Command System

Because of the severe time limitation, the health officer activated the health department’s internal protocol for rapid response and instructed the department duty officer to activate the Department Operations Center (DOC). The Incident Command System (ICS) was initiated by the health department’s public health emergency preparedness and response branch, headed by the DOC director, and finance, operations, logistics, and planning branches were staffed. DOC members were assigned to coordinate onsite TB testing of school staff members and students on December 20; additional DOC members were assigned to coordinate onsite reading of test results on December 23 (Figure).

The DOC identified the following four objectives: 1) complete the testing of all students and staff members on December 20; 2) ensure a safe and secure environment for response staff, school staff, and students; 3) ensure safety precautions were maintained; and 4) establish and maintain situational awareness of clinic operations. Operational priorities identified by the DOC included sufficient staffing for the on-campus health clinic and obtaining sufficient materials for testing. Staffing was secured by pulling a wide cross-section of trained personnel from across the county, facilitated by health department administration, with high school staff members serving under the operations section in the field. Coordination with city and county emergency staff members was facilitated by conference call and onsite liaisons to keep local officials informed.

The public health information officer coordinated press briefings with the school district, county, and city to ensure uniform messaging, and acted as the single point of contact for inquiries. The school pledged full cooperation and mandated all students and staff members to be cleared before returning to school. Written notifications in English and Spanish were sent the same day by the school to advise parents and the school staff of the on-campus clinic.

Through ICS logistics, the DOC worked with the county health care system to acquire sufficient purified protein derivative stocks from internal and external sources to provide tuberculin skin tests for at least 2,000 persons. For students and staff members who were traveling and could not return to have their tests read within the 48-hour timeframe, the department’s public health laboratory coordinated staffing and sufficient reagents to perform 400 interferon-gamma release assays (IGRAs), which do not require a return for reading; limited quantities of reagents precluded its use for all tests.

On Friday, December 20, approximately 55 staff members operated the on-campus clinic from 8 a.m. to 3 p.m. In this 7-hour period, 1,494 persons had tuberculin skin tests performed, and 213 had blood drawn for IGRAs. Ninety-nine persons had a history of a previously positive tuberculin skin test and were instead evaluated by nursing staff for symptoms, with referral for a chest radiograph if history suggested disease.

Over the weekend, the public health laboratory processed the 213 IGRAs as an early indicator. Thirteen (6.1%) were positive for TB, and eight (3.8%) were indeterminate.

On Monday, December 23, tuberculin skin tests results for 1,464 of the 1,494 persons tested were read. A total of 133 (9.1%) had >10mm of induration and were referred for radiographs along with the 13 persons with positive IGRA results.

## Treatment and Follow-up

The disease control branch of the health department continued to coordinate follow-up of pending test results, including among family members and those who visited outside health care providers. The school continued to require that testing and clearance be completed before readmission. A second testing of the 198 original contacts after the window period had elapsed indicated 10 new converters.

Two of the abnormal radiographs were judged severe enough to consider active disease, one with new-onset pleural effusion, and another with a large, potentially cavitary mass. The first person was hospitalized for workup; no other potential etiology was found, and the patient was started on a standard four-drug antituberculosis regimen. The second patient also was started on TB treatment by the public health officer as an outpatient. Neither demonstrated positive smears or culture results for TB, but both demonstrated marked radiographic improvement at the 2-month mark and completed 4 months of TB treatment for presumptive disease. No other cases were found. The remaining abnormal radiographs were judged most consistent with healed TB.

Of the remaining positive tests, the 69 persons with positive results in the initial cohort of 198 were offered 12 weekly doses of isoniazid and rifapentine under directly observed therapy for LTBI because of their perceived greater risk for active TB; 35 accepted treatment, and the remainder were lost to follow-up, refused, or visited their personal medical provider. All treatment regimens were successfully completed. The 146 persons with positive results from the second cohort of 1,806 were offered 6 months of isoniazid for LTBI, of whom 37 accepted treatment. Twenty-two persons from the initial cohort were lost to follow-up; 2,004 persons were evaluated in total.

## Discussion

Testing an entire high school for TB is uncommon, although a similar event occurred at a Colorado school in 2011, where 1,249 persons were screened (1). In that event, the percentage triggering the all-school testing was approximately 35% positive tests from a cohort of 140. In Riverside County, positive TB test rates were expected to range between 10% and 15% in a typical cohort; the 30% rate was considered extraordinary and suggestive of sustained transmission.

The Colorado incident was managed using ICS and 12 separate clinics over 1 month, whereas the Riverside County event demonstrates that the use of ICS can mobilize resources among health departments and their branches in a substantially shorter timeframe. The clearly defined command structure of ICS has been proven to aid resource and information flow and to provide for public communication and coordination with other local agencies (2). The rapid deployment and large-scale operation of the clinic required more resources than the public health department had immediately available; ICS logistics was able to obtain additional resources from other departments countywide, with strong support from county government and department administration. Large-scale testing operations might generate large numbers of positive test results, requiring greater resources and prioritization of labor-intensive LTBI regimens. In Riverside County, the number of nurses available for directly observed therapy was limited; therefore, the 12-dose isoniazid-rifapentine option was reserved for those determined at greater risk because of exposure history.

The presence of mental health staff members onsite was particularly important in providing immediate support to students and school staff members. They provided general support to address fears and anxiety brought on by the event and response activities, as well as to those who received positive test results. Prompt counseling and reassurance helped to allay the health fears of those who tested positive and facilitated their quicker evaluation.

What is already known on this topic?

Tuberculosis (TB) can pose a substantial risk in congregate settings, such as schools, care facilities, and prisons. Little evidence base exists regarding when contact investigations should be rapidly expanded.

What is added by this report?

This report describes use of an Incident Command System (ICS) to rapidly deploy a large-scale TB testing operation (1,806 persons tested in a single clinic within 24 hours) after 59 (29.8%) persons in the initial contact investigation cohort of 198 tested positive. A total of 1,494 persons were screened by tuberculin skin testing, with 133 testing positive (9.1%); 213 persons were screened by interferon-gamma release assay testing, with 13 testing positive (6.1%). A total of 37 persons accepted treatment for latent TB infection, and two secondary active TB cases were presumptively identified and treated.

What are the implications for public health practice?

An ICS should be considered as a management and response tool for large-scale TB screenings that might be warranted by abnormally high TB test conversion rates during an initial contact investigation.

Cooperation with the school and city officials was critical for success. The city provided security, and the school provided space for the testing operations; school nursing staff assisted with reading skin tests and performing symptom screening, and the school administration’s enforcement of clearance before readmission greatly enhanced testing rates. However, the large open area in which testing was performed might have enabled other persons to infer test results based on the next station persons were sent to. Partitioning or standardized stations with uniform patient flow might be required to improve confidentiality in large testing venues.

Effective media relations also were recognized as an opportunity for improvement. During skin testing and reading, the media were kept off-premises. However, the large number of media inquiries to multiple departments might have been better facilitated with the establishment of a joint information center administered through the DOC.

## Figures and Tables

**FIGURE:**
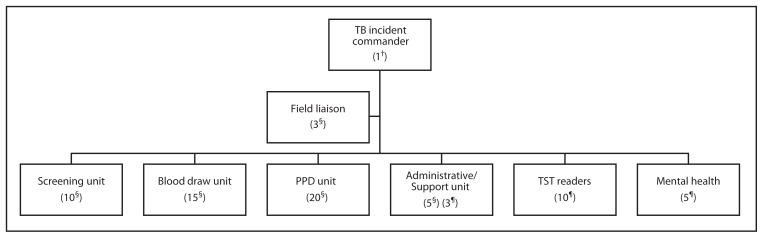
Organization chart showing Incident Command System staffing* for onsite testing (December 20) and results reading (December 23) in response to a tuberculosis (TB) outbreak in a high school — County of Riverside Department of Public Health, California, 2013 **Abbreviations:** PPD = purified protein derivative; TST = tuberculin skin test. * No. of staff members in parentheses. ^†^ December 20 and 23. ^§^ December 20. ^¶^ December 23.
